# Prediction of Hospital Readmission from Longitudinal Mobile Data Streams

**DOI:** 10.3390/s21227510

**Published:** 2021-11-12

**Authors:** Chen Qian, Patraporn Leelaprachakul, Matthew Landers, Carissa Low, Anind K. Dey, Afsaneh Doryab

**Affiliations:** 1 Department of Engineering Systems and Environment, University of Virginia, Charlottesville, VA 22904, USA; cq4sk@virginia.edu; 2 Heinz College of Information Systems and Public Policy, Carnegie Mellon University, Pittsburgh, PA 15213, USA; pleelapr@andrew.cmu.edu; 3 Department of Computer Science, University of Virginia, Charlottesville, VA 22904, USA; qwp4pk@virginia.edu; 4 Department of Medicine, University of Pittsburgh, Pittsburgh, PA 15213, USA; lowca@upmc.edu; 5 Information School, University of Washington, Seattle, WA 98105, USA; anind@uw.edu

**Keywords:** mobile and wearable sensing, data processing, feature extraction, deep learning, patient readmission

## Abstract

Hospital readmissions impose an extreme burden on both health systems and patients. Timely management of the postoperative complications that result in readmissions is necessary to mitigate the effects of these events. However, accurately predicting readmissions is very challenging, and current approaches demonstrated a limited ability to forecast which patients are likely to be readmitted. Our research addresses the challenge of daily readmission risk prediction after the hospital discharge via leveraging the abilities of mobile data streams collected from patients devices in a probabilistic deep learning framework. Through extensive experiments on a real-world dataset that includes smartphone and Fitbit device data from 49 patients collected for 60 days after discharge, we demonstrate our framework’s ability to closely simulate the readmission risk trajectories for cancer patients.

## 1. Introduction

Hospital readmissions are associated with high mortality rate and suffering for both patients and family members, and they cost the US health system billions of dollars. Unplanned patient readmissions following surgery are common in many treatments, with nearly a third of patients being readmitted within 30 days of discharge from the hospital [1]. According to Zafar et al. (2018) [2], up to 80% of readmsissions after cancer surgeries can be prevented if the readmission risk is accurately estimated and predicted. However, predicting who will be readmitted following a surgery is often challenging. Current approaches to readmission risk estimation use patients’ demographics, medical history, and hospital administrative information to estimate a singular risk of readmission in patients at the time of discharge and often categorize most patients as high-risk [3,4]. In addition, traditional measures use static information at the time of discharge and do not take into account other factors in patients’ daily life that may contribute to the increase or decrease of their readmission risk. These factors among others include a patient’s daily behavioral activities that affect their recovery after treatment. Currently, there is no mechanism to objectively measure readmission risk nor is there a method by which readmission risk can be updated over time based on changes in patients’ daily activity and behavior. Such a mechanism would help identify risk acuity ahead of time and provide the opportunity for healthcare providers to act accordingly.

Mobile and wearable devices made it possible to track daily activity and behavior of people seamlessly and unobtrusively [5]. Through the analysis of signals collected from different sensing channels (e.g., GPS for location tracking and pedometer for activity tracking) and data sources (e.g., call and SMS logs), patterns of behavior can be analyzed and used for different purposes such as health promotion [6] and medical diagnosis [7]. A few studies leveraged patients’ activity data collected on Fitbit devices during hospitalization to predict whether or not the patient would be readmitted after discharge [8,9,10]. These studies, in addition to their small scope and duration, only focus on prediction of the readmission status rather than continuous monitoring and prediction of the daily risk.

Our research addresses the challenge of daily readmission risk prediction after the hospital discharge through leveraging the abilities of mobile devices and deep learning models. Recurrent Neural Network strategies such as LSTMs (Long-Short Term Memory) are particularly appropriate for processing sequential data (like daily behavioral data) and have the ability to accurately predict the next states in a model [11,12]. We leverage these capabilities in a probabilistic framework to predict daily progression of readmission risk in the patient population. Our evaluation of the framework using data from mobile phones and Fitbit devices from 49 patients collected over 60 days after discharge from the hospital demonstrate the ability of our framework to closely predict daily readmission risk progressions in cancer patients, minimizing the error between the predicted and actual readmission risk and outperforming classical regression approaches. Our contributions are as follows:

- We develop a new method that incorporates the static medical administration risk assessment value currently used in hospitals as the initial risk probability in an LSTM structure to infer the readmission risk progression trajectory of each patient from their processed behavioral mobile data.
- We develop a new ranking method to evaluate the performance of the generated models. This method particularly contributes to selecting models that more accurately estimate the levels of risk in early days after discharge where patients are at a higher risk of readmission.
- To our knowledge, this is the first approach for prediction of daily readmission risk progression through leveraging mobile data and deep learning frameworks. This framework can contribute to the continuous monitoring of patients outside of hospitals and help clinical professionals to identify patients at risk and act accordingly.

In the following, we first present the related work in readmission prediction and discuss the application of some machine learning and deep learning methods to predict readmission. We then introduce our framework to measure the daily risk probability in an LSTM structure. We describe our designed functions to simulate the daily risk of readmission probabilities and present the evaluation results of the framework on a real-world dataset.

## 2. Background and Related Work

Unplanned readmissions following cancer surgery are common—22–35% of pancreatic surgery patients readmitted within 30 to 90 days of discharge from the hospital [1]. Although some readmissions are unavoidable, an estimated 82% of readmissions after cancer surgery are judged to be potentially preventable [2]. The most common causes for readmission after cancer surgery include infections, gastrointestinal problems, and nutritional deficiency or dehydration [2,13], all of which could be managed on an outpatient basis if detected earlier. Existing risk calculators are based on administrative data available at time of discharge, but surgical recovery continues after hospital discharge. Continuous remote patient monitoring across the transition from hospital to home could allow clinicians to detect subtle changes in patient behavior and physiology that signal increasing readmission risk and to intervene earlier, before complications escalate.

While the period of monitoring after hospital discharge is important, a specific monitoring time window was not yet identified. Douglas et al. [14] found that, while not statistically significant, the probability of readmission decreases over time with the greatest risk of readmission being within the first 30 days after the hospital discharge date. After 30 days, the hazard function curves flatten out; after 60 days, there is a negligible change in the probability of readmission. Carrol et al. [15] demonstrated that there is a significant decrease of mortality for pancreatic adenocarcinoma in the first 60 days, and the risk of death does not change significantly between 60 days and 2 years, which indicates that 60 days may be an important endpoint.

When we followed up with the hospital administration about our studied patient population, they confirmed that none of the patients in our study were hospitalized after 60 days of discharge for complications related to their surgery. As such, the above mentioned studies and observations from our patient population suggest a 60-day period as a solid monitoring time window for patients after hospital discharge.

Significant past work has explored the potential of using machine learning models in readmission prediction [16,17]. For example, an analysis of machine learning techniques for heart failure readmissions by Mortazavi et al. [18] compared three popular machine learning methods—random forest, boosting, and support vector machine—to identify comprehensive factors that contribute to heart failure readmission compared to a baseline of logistic regression. Frizzell et al. [19] also examined readmission prediction in heart failure patients, comparing three machine learning models—tree-augmented naive bayesian network, random forest, and gradient boosting—with traditional statistical methods. The result showed a similar discrimination ability over all methods applied to the same dataset.

The artificial neural network model was used in early readmission prediction for several medical conditions. Aswathi et al. [20] compared the performance of Multilayer Perceptron (MLP) to Logistic Regression for the early prediction of readmission of diabetic patients. They used features derived from patient health records from clinical encounters, including the index admission, discharge details, time in the hospital, diagnosis, and lab results. Maragatham et al. [21] also utilized the electronic health record for early prediction of heart failure using LSTM. This work reported improved performance of LSTM over traditional machine learning methods, including K-Nearest Neighbors, Logistic Regression, Support Vector Machine, and Multi-layer Perceptron. Another study by Reddy et al. [22] also implemented an LSTM structure to predict readmissions in patients with lupus. The results demonstrated improvement in prediction using the LSTM over neural network and logistic regression architecture on longitudinal records.

The previously mentioned studies attempted to predict whether or not the patient would be readmitted during a specific time period (classification). However, these analyses did not capture time until the readmission occurred. Vinzamuri et al. [23] described the development of survival regression, which analyzes the expected duration of time until one or more events of interest occur, for censored data on electronic health records of aggregated lab values for 30-day heart failure readmission, and calculated the hazard probability of readmission for 30, 60, and 90 days readmission as a feature of the prediction. Bussy et al. [24] proposed the use of the time-to-event outcome framework by combining survival analysis together with classification methodology. The work utilized data from sickle-cell disease patients and compared five machine learning classification models and three other survival analysis methods. The study concluded that using survival regression analysis on the problem of early-readmission could yield better performance compared to directly using classification methodology. However, none of these previous studies pursued the use of LSTM to predict readmission based on day-to-day behavioral data from smartphone and personal wearable sensors. Our contributions include developing a new deep learning framework for sequential prediction of readmission risk from behavioral data streams collected from smartphones and wearable devices. In our studies, we derived readmission probability from risk stratification metrics, including HOSPITAL and LACE scores, and then design an LSTM model to predict the daily probability of readmissions.

Only a few studies used data from sources other than patient health records and demographics for readmission prediction. Bae et al. [9] explored the potential of Fitbit data to predict readmission status among cancer patients during hospitalization. The analysis demonstrated that more sedentary behavior during in-hospital recovery contributes to early readmission. Doryab et al. [8] explored the prediction of readmission risk from disruptions in patients’ biobehavioral rhythms in three stages of treatment, namely before surgery, during hospitalization, and after discharge and showed circadian disruptions are predictive of readmission status after discharge. While we leverage the same type of data as this past work, we explore daily readmission risk prediction rather than only readmission status. To our knowledge this is the first deep learning framework for prediction of daily readmission risk from smartphone and wearable data streams. The following sections describe our framework followed by an evaluation using a real-world patient dataset.

## 3. Deep Learning Framework for Prediction of Daily Readmission Risk

Our framework for measuring daily readmission risk is composed of three main components. The first component calculates the daily probability risk from existing hospital administrative information. The second component utilizes the LSTM structure to process sensor data streams as input for readmission prediction, and the third component leverages ranking and statistical methods to measure the performance of the LSTM models in predicting daily risk probability. The following describes each component in details.

### 3.1. Calculating Probabilities of Readmission Risk

Given that the readmission status of the patients is unknown at the time of discharge, calculating daily readmission risk is a challenging task. We address the challenge by utilizing two validated measures called HOSPITAL score and LACE index that are widely used in hospitals to estimate risk of readmission at time of patient discharge [25]. LACE derives its score from **L**ength of stay at the hospital, **A**cuity of the admission, **C**omorbidities, and **E**mergency department visits during the previous 6 months, while HOSPITAL relies on low **H**emoglobin level at discharge, discharge from an **O**ncology service, low **S**odium level at discharge, **P**rocedure during hospital stay, **I**ndex admission **T**ype, number of hospital **A**dmissions during the previous year, and **L**ength of stay. We use these measures to calculate an initial risk probability at the time of hospital discharge. The initial probability is then used in other functions we develop to measure a daily readmission risk.

LACE estimates risk of readmission or death of a patient within 30 days of discharge. The index ranges from 0 to 19, with scores greater than 10 classified as high-risk. HOSPITAL identifies patients at a high risk of potentially avoidable hospital readmission within 30 days of discharge. The HOSPITAL index ranges from 0 to 15, with scores greater than 7 classified as high risk. HOSPITAL and LACE have exhibited good performance in the prediction of readmission risk in several studies [25,26,27]. However, these studies also show that these tools had only moderate discriminative ability, hence additional information is required to improve accuracy [26,27]. These two measures are also static by nature and do not predict the progression of readmission risk over time. However, they can be used as initial risk probabilities for our model.

#### 3.1.1. Initial Risk of Readmission

The initial risk of readmission (the risk on the day after discharge) is generated directly from the LACE and HOSPITAL indices. For example, if a patient receives a LACE score of 0 (i.e., unlikely to be readmitted), their initial probability of readmission is 0. If the patient receives a LACE score of 19 (highest score), their initial probability of readmission is 1. The probability of different scores are calculated linearly. For instance, the readmission probability for a score of 10 is 10/19=0.53.

#### 3.1.2. Final Risk of Readmission

As mentioned in the background section, both existing studies and observations of our patient population suggest that the risk of surgery-related readmission is almost 0 after 60 days of discharge. As such, for nonreadmitted patients, we set the final risk of readmission for surgery to 0 on the 60th day after discharge. For readmitted patients, the risk is set to 1 on the day they were readmitted and dropped to 0 on the 60th day.

#### 3.1.3. Daily Risk of Readmission

**Figure 1:**
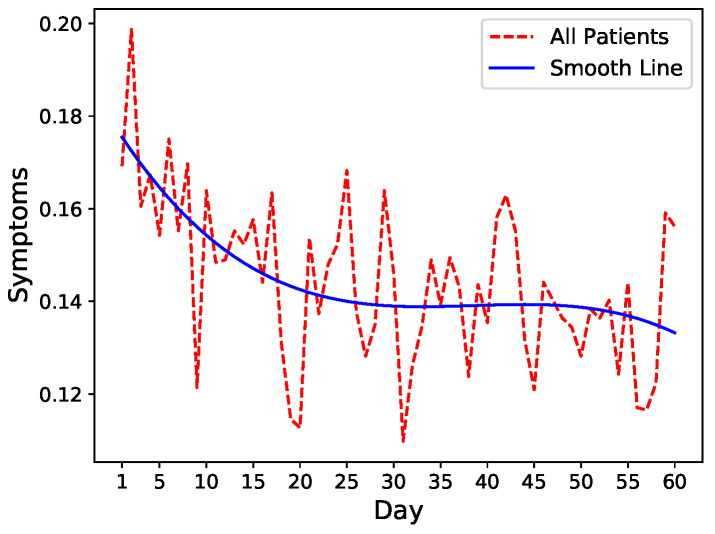
Mean of four symptoms (Pain, Fatigue, Nausea, and Diarrhea) for all patients.

To get an intuition of the readmission risk trends, we leverage fluctuations in symptom levels as a proxy of increase or decrease in readmission risk. We, therefore, run an exploratory analysis on common symptoms including pain, fatigue, nausea, and diarrhea that closely mimic the severity of the patient’s condition after discharge. We calculate the mean of these four symptoms after normalization and visualize the trends combined with spline interpolation for smoothing. As shown in Figure 1, there is a general decrease in symptoms severity in all patients after hospital discharge indicating slow recovery.

**Figure 2:**
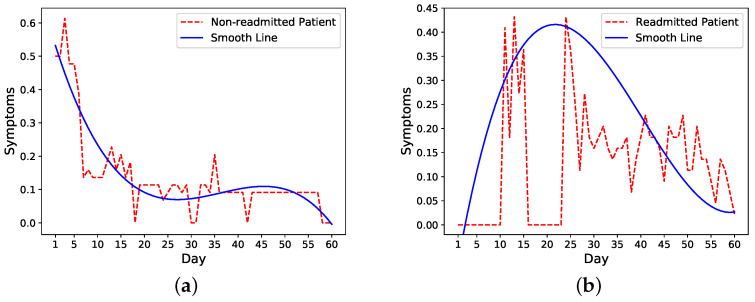
Mean of four symptoms (Pain, Fatigue, Nausea, and Diarrhea) for single patients. (**a**) Nonreadmitted patient. (**b**) Readmitted patient (Readmission day = 16, Second discharge day = 21).

We then look more closely into symptoms trends in readmitted and nonreadmitted patients. The trends of one nonreadmitted patient and one readmitted patient in Figure 2a,b show that for the nonreadmitted patient, the symptoms score decreases from a relatively large value to a small value on the 60th day. For the readmitted patient, on the other hand, the symptoms level peaks around the readmission (day 16) and starts to decrease a few days after the second discharge on day 21.

Based on these observations, we make an assumption that the trend of daily risk probability is similar to the trend of the symptoms score, which means for nonreadmitted patients, the probability would decrease from an initial probability to 0; for readmitted patients, the probability would increase to maximum of 1 on the readmission day and decrease to 0 after the second discharge. We test two cases for readmitted patients. In the first case, we reset the risk probability after the second discharge to the initial probability and decrease it to 0 on day 60. In the second case, we continue with the probability of 1 as the time of second discharge and decrease it to 0 on day 60 following our probability functions. We compare the performance of these two methods to determine the model that more closely follows a patient’s condition after the second discharge.

We then generate daily risk probability from the initial and final probability values. The probability simulates the risk of readmission on a particular day for each patient. The readmission status within *K* days (K=k if patient is readmitted on day *k*, otherwise k=60). We utilize three functions (linear, exponential, logarithmic) to simulate this trend as follows:

(1) $$p_{n}=a\cdot n+b$$

(2) $$p_{n}=a^{n}+b$$

(3) $$p_{n}=log_{a}n+b$$

where *n* is the index of day, $p_{n}$ is the probability at day *n* after discharge, *a* and *b* are two parameters that could be calculated based on probability values of initial and final day.

We also develop the following improved function based on a linear function called weighted linear function:

(4) $$p_{n}=w\times l_{n}+\left(1-w\right)\times p_{n-1},$$

(5) $$w=\frac{4}{{\left(d_{1}-d_{2}\right)}^{2}}\times {\left(n-\frac{d_{1}+d_{2}}{2}\right)}^{2},$$

where *n* is the day index, $p_{n}$ is the probability of readmissions *n* days after discharge, $l_{n}$ is the probability of readmission at day *n* calculated by Equation (1), $d_{1}$ represent start day index, and $d_{2}$ represents the end day index. The range of the weight, *w*, is between 0 and 1. When *w* equals 1, the probability is fully determined by the linear value. When *w* equals 0, the probability is fully determined by the previous day’s value.

**Figure 3:**
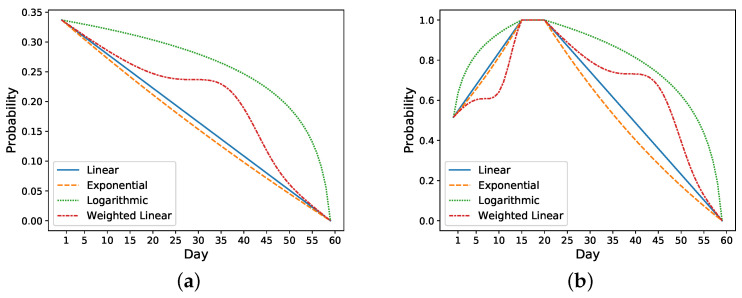
Daily risk probabilities generated by different functions. (**a**) Nonreadmitted patient. (**b**) Readmitted patient (Readmission day = 16, Second discharge day = 21).

Take the previous nonreadmitted and readmitted patients in Figure 2 as an example. For the nonreadmitted patient, the calculated initial risk of readmission based on HOSPITAL or LACE is 0.34 and the final risk is 0 on day 60. Thus, this patient’s daily probability decreases from 0.34 to 0 (see Figure 3a). For the readmitted patient, the readmission occurs on day 16 and the discharge on day 21. Thus, the probability increases from 0.52 on day one to 1 on day 16. Depending on the setting, the risk probability while the patient is in the hospital (i.e., from days 16 to 21) is either set as 1 or decreases from 1 to the initial risk of readmission (i.e., 0.52). After the second discharge, which occurs on day 21, the probability decreases to 0 on day 60. In Figure 3b, the probability is decreased from 1 to 0. Figure 3 shows different approaches for increasing and decreasing the risk: linear, exponential, logarithmic, and weighted linear.

### 3.2. Modeling Approach

We build a two-layer LSTM with a single feed-forward output layer that uses Sigmoid as the activation function to produce the final risk probability. Adam [28] is used as the optimizer during back propagation to adjust the weight of the model to minimize the loss with learning rate 0.01. We use mean-absolute error (see Equation (6)) as the loss function and activate early stopping [29] to avoid overfitting. Based on empirical evidence from experimentation, training ceased when the training loss did not decrease after 50 epochs.

(6) $$MAE=\frac{1}{n}\sum_{i=1}^{n}\left|Y_{i}-{\hat{Y}}_{i}\right|,$$

where *Y* is the actual probability label, $\hat{Y}$ is the prediction, and *n* is the total number of days on which we perform the analysis.

**Figure 4:**
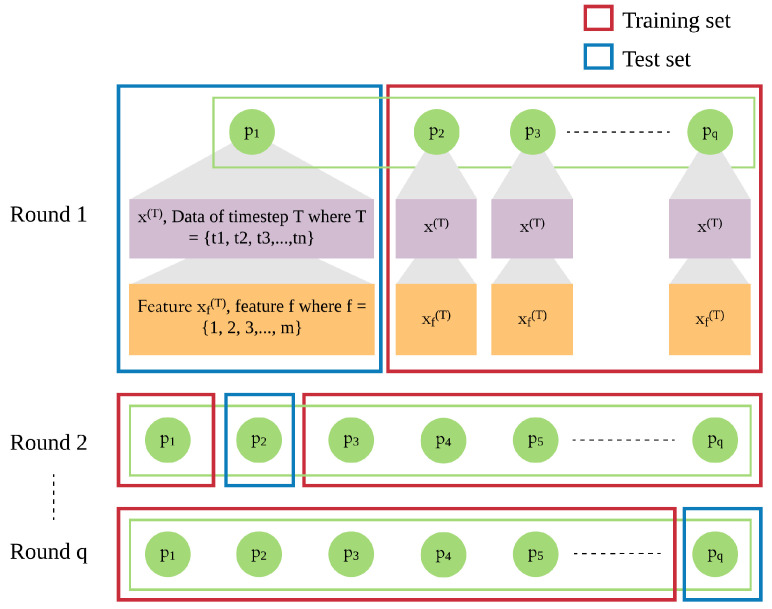
Leave-out-patient-out iteration for model building.

**Figure 5:**
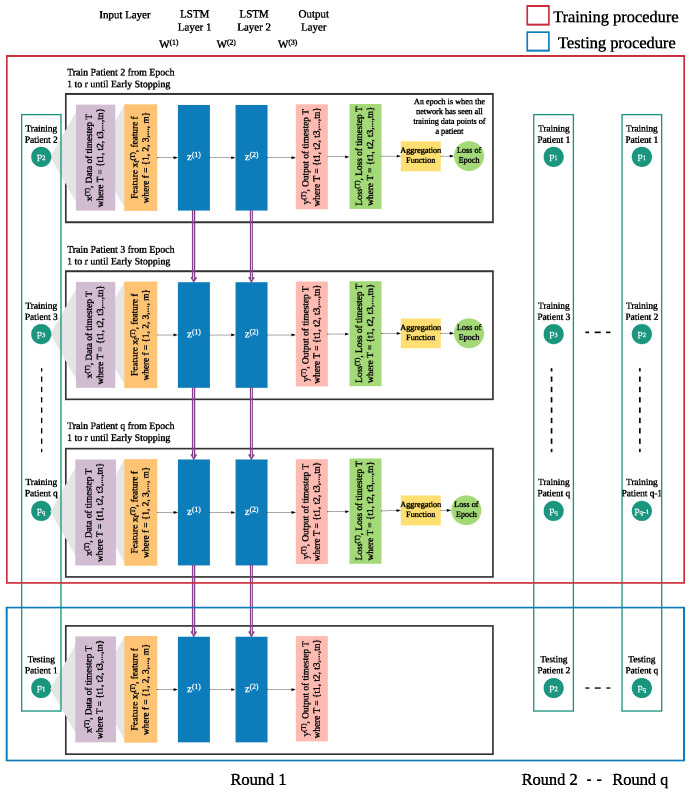
Framework of training and testing procedures.

In addition to the patient’s own data from previous days, we integrate leave-one-patient-out iteration (as shown in Figure 4) into the modeling process to leverage other patients’ data to improve the prediction of daily risk for an individual patient (as shown in Figure 5). We adapt this process in training and testing procedures to build models of patients’ time series data. We then use those models for prediction of risk in the rest of the population, e.g., a new patient. For each patient, one model is trained for multiple rounds.

Each patient *p* has his own sequence of data represented by $x^{\left(T\right)}$, where $T=\{t_{1},t_{2},t_{3},$$\ldots ,t_{n}\}$. Each $x^{T}$ consists of *f* features from 1 to *m*. This sequence of data of patient $p_{1}$ needs to be treated separately from $x^{\left(T\right)}$ of other patients ($p_{2}$ to $p_{q}$), since the sequences of different patients are different. Hence, in each iteration, we leave out the sequence $x^{\left(T\right)}$ of one patient and use $x^{\left(T\right)}$ of the other patients as the training set, until all patients were left out once.

In each round, the trained model is used to produce the risk of readmission prediction $\left({\hat{Y}}_{p}\right)$ against sequence of a patient *p*, where ${\hat{Y}}_{p}=\left\{{\hat{y}}_{p}^{\left(t1\right)},{\hat{y}}_{p}^{\left(t2\right)},{\hat{y}}_{p}^{\left(t3\right)},\ldots ,{\hat{y}}_{p}^{\left(tn\right)}\right\}$ where ${\hat{y}}_{p}^{\left(T\right)}$ is the prediction of input $x_{p}^{\left(T\right)}$ of that will be compared with the actual risk $\left(Y_{p}\right)$ where $Y_{p}=\left\{{\hat{y}}_{p}^{\left(t2\right)},{\hat{y}}_{p}^{\left(t3\right)},\ldots ,{\hat{y}}_{p}^{\left(tn+1\right)}\right\}$ and $y_{p}^{\left(T\right)}$ is the actual risk at timestep *T*.

### 3.3. Using Actual vs. Predicted Probability of Previous Day

**Figure 6:**
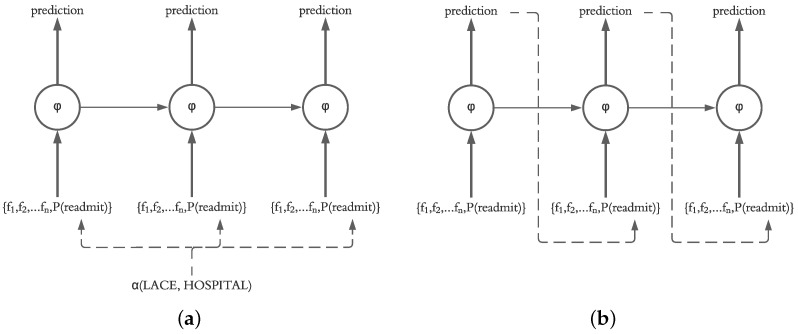
(**a**) Actual probability, i.e., the probability derived from LACE and HOSPITAL scores, as a feature. (**b**) Predicted probability, i.e., the LSTM’s previous output, as a feature.

Our motivation for using an LSTM for prediction of daily readmission risk was based on the ability of LSTM models to sequentially predict the next states in the model. This decision is supported by the fact that the final readmission risk in patients after discharge is unknown, and therefore, a system for prediction of daily readmission risk utilizing our framework should operate based on the predicted probability calculated on a daily basis. However, it is also possible for the system to recalculate the readmission risk of the current and future days based on the known probabilities from previous days. Therefore, in our evaluation, we investigate the impact of injecting the actual probability (calculated using LACE and HOSPITAL) as well as the predicted probability of the previous day (calculated by the LSTM independent of LACE and HOSPITAL scores) into the current state in the LSTM structure, as shown in Figure 6.

### 3.4. Measuring Performance

#### 3.4.1. Ranking Metrics

The performance evaluation of each model is defined by several metrics, including mean squared error (*MSE*) [30] and covariance [31]:

(7) $$MSE=\frac{1}{n}\sum_{i=1}^{n}{\left(Y_{i}-{\hat{Y}}_{i}\right)}^{2},$$

where *Y* is the actual risk of readmission, $\hat{Y}$ is the predicted risk of readmission, and *n* is the total number of days for which we have data across all selected patients.

(8) $$Cov\left(X,Y\right)=\frac{\sum_{i=1}^{n}\left(x_{i}-\bar{x}\right)\left(y_{i}-\bar{y}\right)}{n-1},$$

where *x* and *y* are the independent and dependent variables, $\bar{x}$ is the mean of the independent variable, $\bar{y}$ is the mean of the dependent variable, and *n* is the number of data points in the sample. In our analysis, actual risk of readmission (*Y*) is the independent variable, predicted risk of readmission ($\hat{Y}$) is the dependent variable, and the total number of days for which we have data across all selected patients (*n*) is the number of data points.

Each metric is separated into six submetrics — all patients first *k* (e.g., 20) days, all patients all *K* (e.g., 60) days, readmitted patients first *k* days, readmitted patients all *K* days, nonreadmitted patients first *k* days, and nonreadmitted patients all *K* days. This allowed us to evaluate overall performance, performance for readmitted patients, and performance for nonreadmitted patients separately. The performance matrix $Per{\left(\hat{Y},Y\right)}_{readmitted}$ will consist of ${\hat{y}}_{p}^{\left(T\right)}$, $y_{p}^{\left(T\right)}$, and *n* (the number of patients) for readmitted patients. The calculation for nonreadmitted patients is similar to readmitted patients.

#### 3.4.2. Ranking Process

We define a ranking process to determine which model parameters perform best. To leverage several metrics (MSE and Covariance), we need to accumulate these metrics into one ranking system which can output the top few models. The ranking process is described as follows:

(9) $$Point=\sum_{i=1}^{n}\left(\frac{n_{m}-Rank\left(M_{i}\right)}{n_{m}-1}\right),$$

(10) $$Ranking_{final}=Rank\left(Point\right),$$

where $M_{i}$ are the metrics that are ranked by the ranking order described in Table 1, *n* is the number of metrics, and $n_{m}$ is the number of models that need to be compared. After each metric $M_{i}$ is ranked, the result of the ranking order is denoted by $Rank(M_{i})$. The better performance on that metric will result in a lower $Rank(M_{i})$. All $Rank(M_{i})$ are summed using Equation (9) to calculate the total points, which will be ranked again from largest to smallest to generate the final ranking (i.e., with Equation (10)). $Ranking_{final}$ is used to decide which parameter settings result in the best performance.

### 3.5. Classic Models as Baselines

Given daily readmission risk prediction can be thought of as a regression problem, we compare several classic methods, including Multiple Linear Regression (MLR) [32], Regression Tree (RTF) [33], and Support Vector Regression (SVR) [34], with our LSTM models. Each of these methods uses the same data as inputs, and each method outputs the predicted daily risk probability. Default parameters in the scikit-learn package are used for these algorithms to build baseline models.

#### 3.5.1. Multiple Linear Regression (MLR)

MLR is one of the most common models in predictive modeling. In multiple linear regression, a dependent variable is determined by multiple independent variables and can be written as [32]:

(11) $$\hat{Y}=a_{0}+\sum_{i=1}^{n}a_{i}X_{i},$$

where $\hat{Y}$ is the output of the model, $X_{i}$ is the independent input variables, and $a_{0},a_{1},\cdots ,a_{n}$ are different regression coefficients.

#### 3.5.2. Regression Tree (RT)

RT is an application of decision trees, which is utilized when the predicted outcome is a continuous value or a real number. RT divides the feature space into units, each of which has a specific output. The test data are assigned to different units depending on the features to obtain the corresponding output values. In this study, we employ the Classification and Regression Tree (CART) algorithm [33] to predict daily risk probabilities.

#### 3.5.3. Support Vector Regression (SVR)

SVR is a two-class classification model whose basic model is defined as a linear classifier with the largest spacing in the feature space [34]. The learning strategy of SVR is to maximize the spacing, which ultimately translates into the solution of a convex quadratic planning problem. SVR proved to be an effective tool in real-value function estimation [35].

## 4. Evaluation

### 4.1. Dataset

We use a dataset of smartphones and Fitbit collected from 49 pancreatic patients undergoing abdominal cancer surgery (mean age 65 years, age range 40–82, 43% female, 94% White, 78% married/living as married). This same dataset was used in [8]. Most patients were undergoing surgery for pancreatic cancer (83%) with the remainder undergoing surgery for benign conditions (e.g., pancreatic cysts). The average length of inpatient stay following surgery was 7 days (range 2–22). One-third of patients (34%, n = 18) were readmitted within 60 days of discharge. Readmission to outside facilities was determined by outside hospital records and/or direct patient or caregiver reporting documented in the electronic medical record. There were no significant differences between readmitted and nonreadmitted patients with regard to age (*p* = 0.72), gender (*p* = 0.38), preoperative body mass index (*p* = 0.40), comorbidity (*p* = 0.88), estimated blood loss during surgery (*p* = 0.20), tumor size (*p* = 1.00), or length of hospital stay (*p* = 0.06).

The sensor data were collected using the AWARE framework [36], a mobile application that passively collects behavioral data from smartphones and wearables. AWARE is available for both Android and iOS. The application allows for the collection of a variety of data including activities, calls, heart rate, light, location, messages, screen time, sleep, and step counts. The collected data in our dataset included movement and approximate location of the phone, phone and app use, and call and short message service (SMS) events. The AWARE app also collected patients reports of the severity of 10 symptoms in the range of 0 (the least severe) to 10 (the most severe) once per day. Rated symptoms include pain, fatigue, feeling sad, feeling anxious, feeling hard to concentrate, shortness of breath, numbness, nausea, diarrhea, and sleep disturbances. The Fitbit data included activity in form of the number of steps, sleep, and heart rate.

The dataset also contained the information about the readmission status within 60 days of postoperative discharge. We use this information as ground truth to evaluate the performance of our framework in predicting daily readmission risk.

### 4.2. Sensor Data Processing

#### 4.2.1. Feature Extraction

From the data streams, a set of common statistical features such as min, median, mean, max, and standard deviation, as well as more complex behavioral features such as movement patterns, travel distance, and duration of activities are extracted. We also extracted deviation features, i.e., the difference between the feature value on a particular day and the average value of that feature in the past X-day window (here 7 days). In total, before applying the filtering procedure described in Section 4.4.1, we collected 263 features. The specific procedure for extracting features from these sources can be found in [37]. The examples of sensor features are shown in Table 2.

#### 4.2.2. Handling Missing Values

We tested multiple methods to handle missing values for our analysis. The following procedure was decided based on the best performance results:

1. Remove features (columns in the dataset) that miss more than 80% of the values.
2. Remove features with variance less than 0.3.
3. Impute the missing values in the remaining feature cells following the imputation method of Moving Average Algorithm that first uses the moving average of the previous *N* values (N≥1) (if they are nonempty) to replace the missing values for each patient and each feature. The algorithm then replaces the remaining missing cells with a constant value (−1) that is different from the feature values.

#### 4.2.3. Normalization

We normalize all features to be used in the analysis by using MinMaxScaler in the scikit-learn package with the range 0 to 1 as this is a prerequisite of using the LSTM method. Each feature is scaled within the range of all patients.

### 4.3. Model Parameters and Settings

In addition to the structure of LSTM, we examine a set of parameters and settings to find the optimal performing model.

#### 4.3.1. Input Data Source

We build the LSTM models using different combinations of three data sources—sensor features (i.e., features extracted from mobile sensing data), symptoms features (i.e., the level of symptoms reported by patients), and deviation features (i.e., features calculated from the difference between the current day’s feature value and the past *t* days). To vary the models, we use different combinations of multiple data sources when concatenating the data. Based on different combinations, we could decide whether or not to use sensor features, symptoms features, and deviation features.

#### 4.3.2. Data Range

The range of data used in modeling has a tremendous effect on the results. In our approach to predict daily readmission risk from passive sensing data, we build models using four settings: (1) including data after the surgical operation until the day of readmission (in readmitted patients) or 60 days after discharge (in nonreadmitted patients), (2) including data after discharge until the day of readmission (in readmitted patients) or 60 days after discharge (in nonreadmitted patients), (3) excluding data points after readmission (in readmitted patients), and (4) including full range data up to 60 days for all patients (both readmitted and nonreadmitted). Our numerous tests showed that setting 4 provides the best performing results.

#### 4.3.3. Initial and Daily Probability Calculation

We utilize HOSPITAL and LACE scores and the average of HOSPITAL and LACE scores to measure initial readmission risk probabilities. We also apply four functions (linear, exponential, logarithmic, weighted linear) as described in Section 3.1 to calculate daily readmission risk probabilities.

#### 4.3.4. LSTM Parameters

The design of the LSTM structure greatly affects the prediction of the models. Hence, we adjust the architecture of the LSTM to build different models for performance adjustment and comparison. As described in Section 3.2, the parameters for generating LSTM model consist of number of layers, enabling early stopping callback, adjusting number of hidden LSTM unit, loss function, activation function on both LSTM layers and output layer. In our evaluation, we set these parameters as follows:

- The default number of hidden layers is two. However, we build models with one and two layers.
- The early stopping callback will stop the training if the training loss is not decreased after 50 epochs.
- The number of hidden units is adjusted by the number of features. If the adjustment of hidden units is disabled, the number is set to use the total number of features as the count for hidden units.
- Mean absolute error is used as the loss function.
- The activation functions are tanh and sigmoid at hidden layer and output layers, respectively.

### 4.4. Results

Applying the above settings yields several models. For each of these models, we assess the impact of including the date index as an additional feature. We examine this feature separately with the cautious assumption that date is a stable piece of information that may greatly influence the performance of the models.

**Figure 7:**
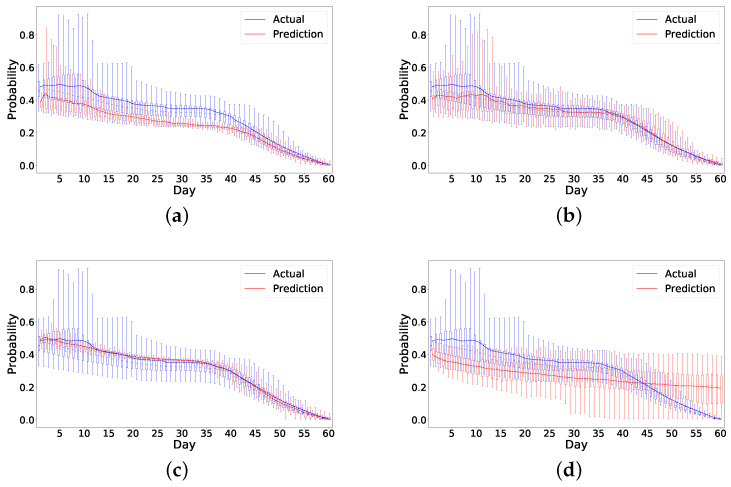
Results for all patients based on default settings. (**a**) Previous actual probability as a feature with date. (**b**) Previous actual probability as a feature without date. (**c**) Previous predicted probability as a feature with date. (**d**) Previous predicted probability as a feature without date.

We present the box plot of actual probability and predicted probability for all patients on each day (shown in Figure 7). This helps us observe the overall performance of the different models.

The models in Figure 7 and Table 3 are the best performing models for the respective settings. Using the previous day’s actual probability produces the closest trend to the actual readmission probability trend line (shown in Figure 7a,b). In this setting, the date index becomes redundant as previous day’s actual probability offers enough information. Using date index in the predicted probability model, however, greatly influences the performance leading the predicted daily probabilities to be closer to the actual probabilities.

#### 4.4.1. Optimizing the Prediction Trend

Although we obtain a good performance through injection of the previous day’s actual probability into the LSTM structure, we are more interested in optimizing models that are independent of this information (i.e., those that operate only based on the predicted probability from previous days). The model without access to the previous day’s actual probability more closely resembles the real-world setting. As such, we choose the model using the previous day’s predicted probability with the date index included as a feature (shown in Figure 7c) as our baseline for further optimization and comparison. Given that this model performs poorly on predicting the daily risk of readmitted patients (See Table 3), we vary different settings to specifically optimize the performance for this group of patients:

- **Imputation with previous *k* days**. We build models with *k* previous days (*k* = 0 to 5) and observe that this impacts the performance of the models. This indicates that closeness in the range of feature values and the patient status is related to these values on previous days. The best performance is observed in the model that uses the average value of the previous two days’ values to impute the missing values (Table 4).
- **Features Filtering**. Removing the features when the first day’s value is missing improves the model’s predictions drastically. This indicates that the first day of data is an important input for the LSTM to initialize the model. Without this input, the feature would act as noise that disturbs the training process (Table 4).
- **Input Data Sources**. Our analysis demonstrates that models that include all sensor, symptoms, and deviation data are more accurate and provide better performance than models that include only a subset of these sources (Table 5). We also find that the performance of the model with only sensor data is better than the model with only symptoms data. This demonstrates that sensor data may play an important role in predicting readmission probabilities.
- **Initial and Daily Probability**. Using an average of HOSPITAL and LACE scores as the initial probability score yields a better performance than does using either score alone. We also observe that the predicted trend lines closely follow the actual probabilities when an exponential function is used to calculate the daily risk of readmission. We also observe that restarting the probability for readmitted patients to the initial probability after the second discharge increases the covariance between the actual and predicted values. The model using the exponential function could simulate the trend of readmission risk appropriately in all patients. (Table 6).

**Figure 8:**
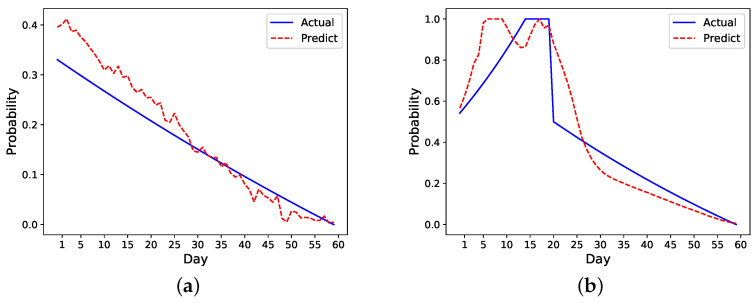
Best model after setting optimization. (**a**) Nonreadmitted patient. (**b**) Readmitted patient (Readmission day = 16, Second discharge day = 21).

Figure 8 demonstrates the performance of the best model, after optimization, at an individual level on our sample patients. We find that this model slightly overestimates the risk of both readmitted and nonreadmitted patients. This is acceptable because our framework aims to detect the risk of readmission and make necessary alerts. As such, a higher predicted probability than the actual probability for readmitted patients is allowable. This model could accurately predict the trend of readmission risk.

#### 4.4.2. Comparison with Classic Models

The LSTM model that used the average of LACE and HOSPITAL as the initial probability and exponential function to calculate the daily risk and was applied on the dataset including sensor, symptoms, and deviation features provided the closest predictions to the actual probability using 2-day imputation and empty feature filtering. We use this model to compare with the outputs of some classic machine learning approaches. As demonstrated in Table 7, LSTM performs better than all classic models. Moreover, by their nature, these classic regression models only use the data on a day by day basis without utilizing the sequential aspect of the prediction including the previous day’s predicted probability. As such, these models only provide prediction of the risk on a certain day and cannot be used as forecast models without additional ground truth.

## 5. Discussion

We designed a deep learning framework based on LSTM to predict the daily risk of readmission in patients after hospital discharge. By using a dataset of smartphone sensors and Fitbit logs, we extracted different features from sensors and symptoms and also calculated the deviation of those features from previous days. Our results showed that all of these features are helpful in predicting the risk of readmission.

Most existing studies predict the readmission status as a classification problem and only a few predict the daily status of patients. By utilizing different functions including linear, exponential, logarithmic and weighted linear function, we designed a method to simulate the daily risk of readmission on patients; our results demonstrated the feasibility of this approach. Compared with classic models, the LSTM could better extract useful information and make full use of the predicted values of previous days, which improves performance.

We also develop a ranking algorithm as part of the framework to evaluate the performance of different models. As there are metrics evaluating the first few days’ performance, this ranking mechanism in particular helps identify models that more accurately predict the early risk of readmission in patients within the first few days of hospital discharge. As the number of readmissions are higher within the first seven days after discharge, the ability to identify patients at risk is of utmost importance. The model chosen by this ranking mechanism shows a good performance on prediction as shown in Figure 8. Specifically, it overestimates the risk in the readmitted patient a few days before the readmission happens, which is ideal as it can raise alarm to clinicians or caregivers to act ahead of time. Although the risk is also overestimated for the nonreadmitted patient, the error margin is small compared to the readmitted patient. In Figure 7 and Figure 8, it also appears that all the models have higher errors in the first few days than in later days. We believe this could be due to a lack of data in the beginning as we only have 49 patients for analysis. This problem is similar to the cold start problem [38] and could be solved if we collect enough data in the future.

While our initial evaluation showed the feasibility of our deep learning framework to predict the daily risk of readmission for patients after cancer surgery, more evaluations on different datasets are needed to determine its practical use in the real world. Such a system could capture the status of the patient outside of the hospital by processing and analyzing data from personal devices without disturbing their life, determine their readmission risk on a daily basis and notify medical professionals about patients at potential risk of readmission (e.g., risk above a particular threshold) while there are still opportunities to intervene.

A large part of our framework addresses the challenge of measuring the actual daily risk to evaluate the performance of the models and has less focus on optimizing the LSTM settings that greatly impact the performance results. Also, as our LSTM models may slightly overestimate or underestimate the risk of readmission, our future work will focus on improving the structure of LSTM network to address this issue. Finally, our work used a real-world dataset from patients after cancer surgery and obtained a good performance. Future steps should explore whether this framework is as generalizable to other readmission problems due to diseases such as heart failure.

Although we develop and evaluate this framework in the context of readmission risk assessment, we believe our methods can be generalized and used in similar applications for risk assessment. Our future plans involve further evaluations on larger patient datasets as well as adjustments and refinements of our framework to be evaluated on datasets in other application domains including climate change, weather forecast, and operation research.

## 6. Conclusions

We designed a deep learning framework based on LSTM to predict and simulate the daily risk of readmission for patients after cancer surgery. We developed new methods to estimate the initial and daily readmission probabilities in the LSTM structure. We also developed a ranking method to evaluate the performance of numerous models generated through the framework. Our evaluation of the framework using a dataset of mobile behavioral data from cancer patients showed the ability of the framework to generate and identify models that best predict the readmission trajectories for all patients after hospital discharge. Future studies are needed to further demonstrate the feasibility of this framework in continuous assessment of readmission risk, as well as to evaluate its potential in predicting risk in other application domains.

## Figures and Tables

**Table 1:** Ranking criteria for each metric.

Metrics	MSE	Covariance
Overall (First 20 days & All 60 days)	Smallest to Largest	Largest to Smallest
Readmitted (First 20 days & All 60 days)	Smallest to Largest	Largest to Smallest
Non-readmitted (First 20 days & All 60 days)	Smallest to Largest	Largest to Smallest

**Table 2:** Examples of sensor features.

Features	Description
Activitity	Number of activities, count of activity changes
Call	Number/Duration of incoming/outgoing calls
Heart Rate	Min/max/mean/total of heart rates, min/max/mean/totoal of absolute/positive/negative changes
Light	Min/max/mean/median/standard deviation of illuminance(lux)
Location	Variance/entropy of location, number of visits/time spend at different clusters
Message	Number of incoming/outgoing messages
Screen	Min/max/mean/total length of interaction periods,
Sleep	Min/max/mean length of asleep/awake/efficiency/restless periods
Step	Min/max/mean/total length of active/sedentary periods, total number of steps

**Table 3:** Results of models with default settings (NR: Nonreadmitted patients; R: Readmitted patients).

Model	MSE						Covariance						Point
	First 20 Days			All 60 Days			First 20 Days			All 60 Days			
	All	NR	R	All	NR	R	All	NR	R	All	NR	R	
Actual_prob_without_date	0.01	0.00	0.04	0.01	0.00	0.02	0.02	0.00	0.02	0.03	0.02	0.04	12.00
Actual_prob_with_date	0.02	0.01	0.06	0.01	0.00	0.04	0.01	0.00	0.02	0.02	0.01	0.03	7.67
Predicted_prob_with_date	0.03	0.01	0.08	0.02	0.01	0.05	0.00	0.00	0.00	0.02	0.01	0.02	3.33
Predicted_prob_without_date	0.04	0.02	0.10	0.05	0.04	0.08	0.00	0.00	0.01	0.01	0.01	0.02	1.00

**Table 4:** Results of models with different settings of data processing (NR: Nonreadmitted patients; R: Readmitted patients).

Model		MSE						Covariance						Point
		First 20 Days			All 60 Days			First 20 Days			All 60 Days			
		All	NR	R	All	NR	R	All	NR	R	All	NR	R	
Imputation with Previous k Days	2	0.03	0.01	0.06	0.02	0.01	0.04	0.00	0.00	0.00	0.02	0.02	0.03	8.80
	5	0.03	0.01	0.09	0.02	0.00	0.05	0.00	0.00	0.00	0.02	0.02	0.02	7.20
	1	0.15	0.16	0.12	0.09	0.09	0.07	0.00	0.00	0.00	0.03	0.03	0.04	5.60
	3	0.09	0.08	0.09	0.07	0.07	0.07	0.00	0.00	0.00	0.02	0.02	0.03	5.60
	4	0.15	0.16	0.12	0.09	0.09	0.07	0.00	0.00	0.00	0.03	0.03	0.04	5.60
	0	0.16	0.17	0.13	0.10	0.11	0.08	0.00	0.00	-0.01	0.03	0.03	0.04	3.20
Features Filtering	Yes	0.03	0.01	0.06	0.02	0.01	0.04	0.00	0.00	0.00	0.02	0.02	0.03	11.00
	No	0.13	0.14	0.09	0.10	0.12	0.08	0.00	0.00	0.00	0.02	0.02	0.03	1.00

**Table 5:** Results of models with different settings of input data sources (Sen: Sensor Features; Dev: Deviation Features; Sym: Symptoms Features; NR: Nonreadmitted patients; R: Readmitted patients).

Model		MSE						Covariance						Point
		First 20 Days			All 60 Days			First 20 Days			All 60 Days			
		All	NR	R	All	NR	R	All	NR	R	All	NR	R	
Input Data Sources	All	0.03	0.01	0.06	0.02	0.01	0.04	0.00	0.00	0.00	0.02	0.02	0.03	10.00
	Sym + Dev	0.04	0.03	0.06	0.05	0.05	0.04	0.01	0.01	0.00	0.02	0.01	0.03	6.60
	Sen + Dev	0.03	0.02	0.07	0.03	0.02	0.07	0.00	0.00	0.00	0.02	0.02	0.02	6.60
	Sen only	0.12	0.12	0.12	0.08	0.08	0.09	0.00	0.00	0.00	0.03	0.02	0.04	4.60
	Sym only	0.06	0.04	0.10	0.04	0.03	0.07	0.00	0.00	0.00	0.02	0.01	0.02	4.20
	Sen+Sym	0.06	0.02	0.15	0.03	0.01	0.09	0.00	0.00	0.00	0.01	0.01	0.02	4.00

**Table 6:** Results of models with different settings of generating probability (WL: Weighted Linear Function; Exp: Exponential Function; Lin:Linear Function; Log:Logarithmic Function; ini: Initial Probability; NR: Nonreadmitted patients; R: Readmitted patients).

Model		MSE						Covariance						Point
		First 20 Days			All 60 Days			First 20 Days			All 60 Days			
		All	NR	R	All	NR	R	All	NR	R	All	NR	R	
Initial Probability	Average	0.03	0.01	0.06	0.02	0.01	0.04	0.00	0.00	0.00	0.02	0.02	0.03	9.00
	LACE	0.03	0.01	0.08	0.02	0.00	0.05	0.00	0.00	0.00	0.02	0.02	0.03	8.50
	HOSPITAL	0.16	0.17	0.14	0.10	0.11	0.08	-0.01	0.00	0.00	0.02	0.01	0.03	0.50
Daily Probability	Exp (ini)	0.12	0.11	0.12	0.06	0.05	0.09	0.01	0.00	0.01	0.04	0.03	0.06	8.14
	WL(1)	0.11	0.14	0.05	0.08	0.10	0.05	0.00	0.00	0.00	0.04	0.03	0.05	7.86
	WL(ini)	0.03	0.01	0.06	0.02	0.01	0.04	0.00	0.00	0.00	0.02	0.02	0.03	7.71
	Lin(1)	0.18	0.24	0.04	0.14	0.18	0.04	0.01	0.00	0.00	0.05	0.03	0.08	7.29
	Exp(1)	0.07	0.00	0.23	0.05	0.00	0.16	0.00	0.00	0.00	0.02	0.02	0.04	6.86
	Log(ini)	0.14	0.16	0.10	0.13	0.12	0.13	0.01	0.00	0.01	0.02	0.01	0.03	4.57
	Lin(ini)	0.20	0.24	0.12	0.21	0.23	0.17	0.00	0.00	0.00	0.03	0.03	0.05	3.14
	Log(1)	0.12	0.03	0.35	0.10	0.02	0.29	0.00	0.00	0.00	0.01	0.01	0.01	2.43

**Table 7:** Result of different models (NR: Nonreadmitted patients; R: Readmitted patients).

Model	MSE						Covariance						Point
	First 20 Days			All 60 Days			First 20 Days			All 60 Days			
	All	NR	R	All	NR	R	All	NR	R	All	NR	R	
**LSTM**	**0.12**	**0.11**	**0.12**	**0.06**	**0.05**	**0.09**	**0.01**	**0.00**	**0.01**	**0.04**	**0.03**	**0.06**	**9.33**
MLR	0.13	0.16	0.06	0.13	0.14	0.08	0.00	0.00	0.00	0.02	0.02	0.04	5.67
RT	0.16	0.19	0.10	0.15	0.16	0.12	0.00	0.00	0.00	0.03	0.02	0.04	4.67
SVR	0.12	0.14	0.06	0.13	0.15	0.09	0.00	0.00	0.00	0.02	0.02	0.03	4.33

## Data Availability

The data presented in this study are available on request from the corresponding author. The data are not publicly available due to privacy restrictions.

## Abbreviations

The following abbreviations are used in this manuscript:

LSTM	Long Short-Term Memory
SMS	Short Message Service
GPS	Global Positioning System
MSE	Mean Squared Error
